# The Cell-Cycle Regulatory Protein p21^CIP1/WAF1^ Is Required for Cytolethal Distending Toxin (Cdt)-Induced Apoptosis

**DOI:** 10.3390/pathogens9010038

**Published:** 2020-01-02

**Authors:** Bruce J. Shenker, Lisa M. Walker, Ali Zekavat, Robert H. Weiss, Kathleen Boesze-Battaglia

**Affiliations:** 1 Department of Pathology, University of Pennsylvania School of Dental Medicine, Philadelphia, PA 19104, USA; 2 Department of Medicine, University of California at Davis School of Medicine, Sacramento, CA 95616, USA; 3 Department of Biochemistry, University of Pennsylvania School of Dental Medicine, Philadelphia, PA 19104, USA; battagli@upenn.edu

**Keywords:** *Aggregatibacter actinomycetemcomitans*, cytolethal distending toxin, lymphocytes, apoptosis, virulence

## Abstract

The *Aggregatibacter actinomycetemcomitans* cytolethal distending toxin (Cdt) induces lymphocytes to undergo cell-cycle arrest and apoptosis; toxicity is dependent upon the active Cdt subunit, CdtB. We now demonstrate that p21^CIP1/WAF1^ is critical to Cdt-induced apoptosis. Cdt induces increases in the levels of p21^CIP1/WAF1^ in lymphoid cell lines, Jurkat and MyLa, and in primary human lymphocytes. These increases were dependent upon CdtB’s ability to function as a phosphatidylinositol (PI) 3,4,5-triphosphate (PIP3) phosphatase. It is noteworthy that Cdt-induced increases in the levels of p21^CIP1/WAF1^ were accompanied by a significant decline in the levels of phosphorylated p21^CIP1/WAF1^. The significance of Cdt-induced p21^CIP1/WAF1^ increase was assessed by preventing these changes with a two-pronged approach; pre-incubation with the novel p21^CIP1/WAF1^ inhibitor, UC2288, and development of a p21^CIP1/WAF1^-deficient cell line (Jurkat^p21−^) using clustered regularly interspaced short palindromic repeats (CRISPR)/cas9 gene editing. UC2288 blocked toxin-induced increases in p21^CIP1/WAF1^, and Jurkat^WT^ cells treated with this inhibitor exhibited reduced susceptibility to Cdt-induced apoptosis. Likewise, Jurkat^p21−^ cells failed to undergo toxin-induced apoptosis. The linkage between Cdt, p21^CIP1/WAF1^, and apoptosis was further established by demonstrating that Cdt-induced increases in levels of the pro-apoptotic proteins Bid, Bax, and Bak were dependent upon p21^CIP1/WAF1^ as these changes were not observed in Jurkat^p21−^ cells. Finally, we determined that the p21^CIP1/WAF1^ increases were dependent upon toxin-induced increases in the level and activity of the chaperone heat shock protein (HSP) 90. We propose that p21^CIP1/WAF1^ plays a key pro-apoptotic role in mediating Cdt-induced toxicity.

## 1. Introduction

The cytolethal distending toxin (Cdt) is a putative virulence factor that is produced by a wide range of human pathogens capable of colonizing mucocutaneous tissue, resulting in disease characterized by persistent infection and inflammation (reviewed in References [1,2]). In general, Cdts are heterotrimeric complexes encoded by an operon of three genes designated *cdtA*, *cdtB*, and *cdtC* which encode three polypeptides: CdtA, CdtB, and CdtC with molecular masses of 23–30, 28–32, and 19–20 kDa, respectively [3,4,5,6,7,8,9,10,11,12,13]. Analyses of subunit structure and function indicate that the heterotrimeric holotoxin functions as an AB_2_ toxin; the cell binding unit (B) is responsible for toxin association with the cell surface and is composed of subunits CdtA and CdtC. These subunits deliver the active subunit (A), CdtB, to intracellular compartments. Cdt binding and CdtB internalization are both dependent upon toxin binding to target cell cholesterol in the context of cholesterol-rich membrane microdomains (reviewed in Reference [14]).

Cdt B internalization leads to irreversible cell-cycle arrest and eventually apoptotic cell death. These toxic effects were originally attributable to CdtB’s ability to function as a DNase, thereby causing DNA damage which in turn leads to G2/M arrest and death [9,15,16,17,18,19,20,21,22,23]. Over the past several years, our studies suggested an alternative paradigm to account for *Aggregatibacter actinomycetemcomitans* Cdt-mediated toxicity which is based upon a novel molecular mode of action for CdtB. In this regard, we demonstrated that, in addition to exhibiting DNase activity, CdtB is a potent lipid phosphatase capable of converting the signaling lipid phosphatidylinositol (PI)-3,4,5-triphosphate (PIP3) to PI-3,4-diphosphate [24,25,26,27,28]. Moreover, our investigations demonstrated that the ability of CdtB to function as a PIP3 phosphatase enables this toxin subunit to intoxicate cells via blockade of the PI-3K signaling pathway. Indeed, we demonstrated that the toxic effects of Cdt on lymphocytes, macrophages, and mast cells results in PI-3K signaling blockade characterized by decreases in PIP3, leading to concomitant reductions in the phosphorylation status of downstream targets: Akt and GSK3β. Additionally, we demonstrated that the induction of both G2/M arrest and apoptosis is dependent upon CdtB-mediated PI-3K blockade. 

In order to more accurately define the molecular mechanisms that link CdtB-mediated PI-3K blockade with G2/M arrest and apoptosis, we investigated the role of the cyclin-dependent kinase inhibitor known as CDK-interacting protein 1 (Cip1) and wild-type p53-activated fragment 1 (WAF1) (p21^CIP1/WAF1^). P21^CIP1/WAF1^ was originally identified as a negative regulator of the cell cycle, as well as a tumor suppressor. However, recent studies demonstrated additional functions for p21^CIP1/WAF1^ that are associated with regulation of a number of cellular processes including cell differentiation, migration, senescence, and apoptosis [29,30,31,32,33]. Thus, it is not surprising that several investigators demonstrated an association between p21^CIP1/WAF1^ expression and exposure to Cdt [16,34,35,36,37]. It should be noted, however, that these studies did not provide any information as to whether the p21^CIP1/WAF1^ levels were mechanistically linked to and/or required for Cdt toxicity. In this study, we investigated the relationship between lymphocyte exposure to *A. actinomycetemcomitans* Cdt, altered p21^CIP1/WAF1^ levels, and induction of toxicity. We now report that Cdt-treated human lymphocytes exhibit dose-dependent increases in levels of p21^CIP1/WAF1^ and the chaperone HSP90 within 4–16 h of exposure to the toxin. To study the biologic consequence of these increases, we employed a two-pronged approach to modify the ability of Cdt to alter expression of p21^CIP1/WAF1^: gene editing and pharmacologic intervention. Additionally, these interventions were assessed for their ability to alter cell susceptibility to Cdt toxicity. Our results indicate a requisite role for p21^CIP1/WAF1^ in Cdt-induced apoptosis. 

## 2. Results

### 2.1. Cdt Induces Elevations in Lymphocyte Levels of p21^CIP1/WAF1^

**Figure 1:**
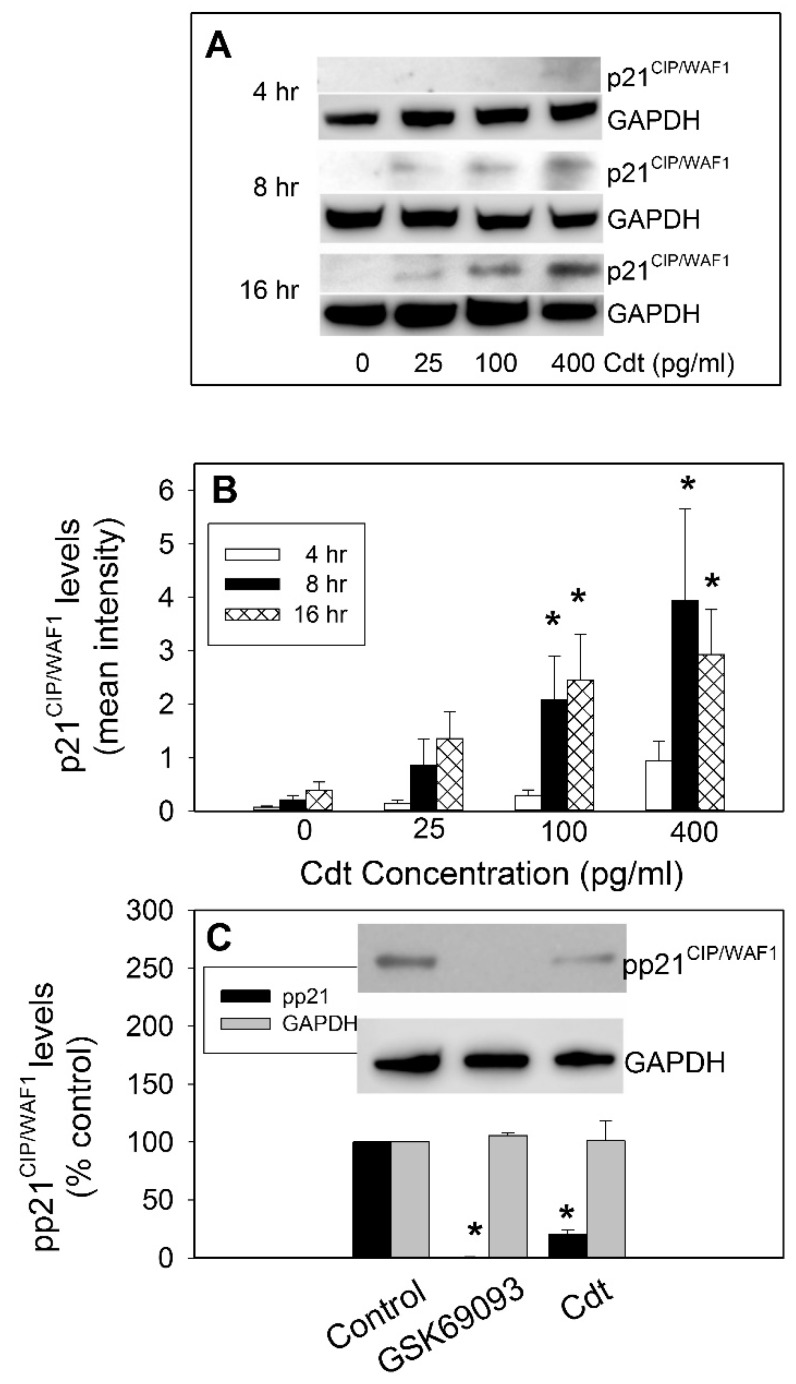
Effect of cytolethal distending toxin (Cdt) on p21^CIP1/WAF1^ levels in Jurkat cells. Jurkat cells were exposed to 0–400 pg/mL Cdt for 4, 8, and 16 h; cells were then harvested, and extracts were fractionated by SDS-PAGE and analyzed by Western blot for the presence of p21^CIP1/WAF1^. Panel (**A**) shows representative Western blots of p21^CIP1/WAF1^ for cells treated with each dose of toxin at 4, 8, and 16 h; glyceraldehyde 3-phosphate dehydrogenase (GAPDH) served as a gel loading control. Panel (**B**) shows the results from multiple blots which were analyzed by digital densitometry; the value of relative intensity obtained from digital densitometry is presented as the mean ± standard error of the mean (SEM) of four experiments. Panel (**C**) shows the relative levels of pp21^CIP1/WAF1^ in cells treated with 50 pg/mL Cdt or 0.5 μM GSK690693 for 16 h expressed as a percentage observed in control (medium only) cells. A representative blot is shown along with compiled results from three experiments: results are expressed as the percentage (mean ± SEM) of pp21^CIP1/WAF1^ observed in control cells; * indicates statistical significance (*p* < 0.05) when compared to untreated cells.

Cdt derived from *A. actinomycetemcomitans*, *Haemophilus ducreyi*, and *Helicobacter hepaticus* were shown to induce increases in p21^CIP1/WAF1^ within 24–48 h in several cell lines including fibroblasts, lymphocytes, enterocytes, and hepatocytes [16,34,35,36,37,38]. Likewise, we now demonstrate that *A. actinomyetemcomitans* Cdt induces increases in p21^CIP1/WAF1^ levels in Jurkat cells in a time- and dose-dependent manner. Jurkat cells were treated with varying amounts of Cdt (0–400 pg/mL) for 4, 8, and 16 h and then analyzed by Western blot to assess total p21^CIP1/WAF1^ levels (Figure 1A,B). Analysis indicates that a small, but consistent, increase in p21^CIP1/WAF1^ was detected within 4 h in cells exposed to the highest concentration of Cdt (400 pg/mL). Following an 8-h exposure, significant increases of nine- and 18-fold were observed in cells exposed to 100 and 400 pg/mL Cdt, respectively. After a 16-h exposure, the relative levels of p21^CIP1/WAF1^ remained elevated; cells treated with 25, 100, and 400 pg/mL Cdt exhibited three-, six-, and seven-fold increases, respectively.

**Figure 2:**
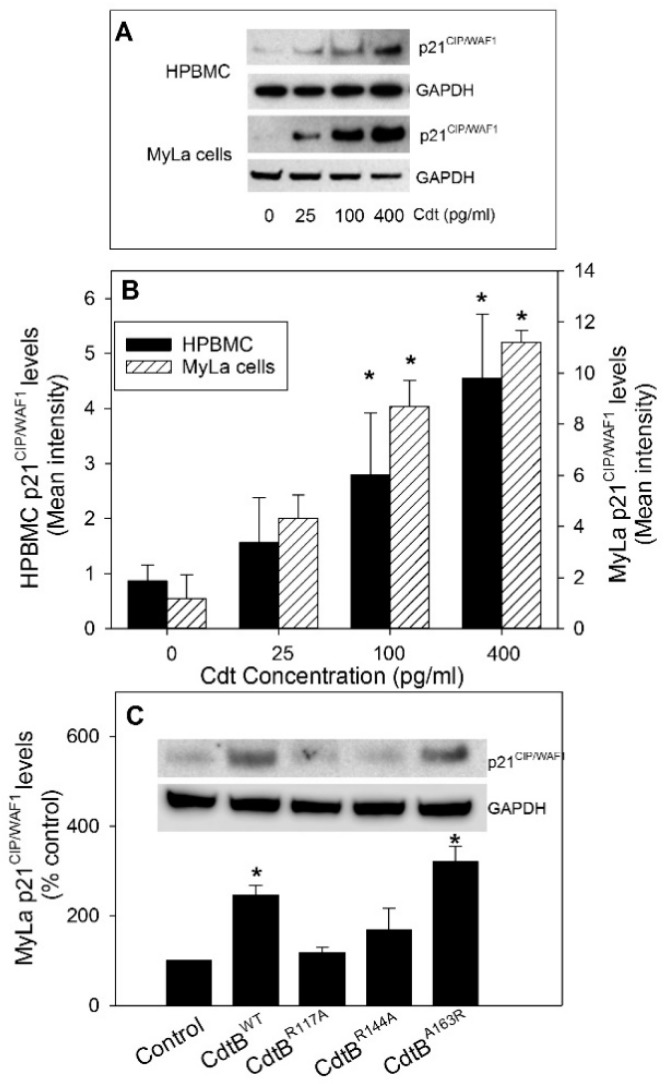
Effect of Cdt on p21^CIP1/WAF1^ levels in primary human lymphocytes (HPBMCs) and MyLa cells. HPBMC and MyLa cells were treated with 0–400 pg/mL Cdt for 16 h. The cells were harvested, and extracts were fractionated by SDS-PAGE and analyzed by Western blot for the presence of p21^CIP1/WAF1^. Panel (**A**) shows representative Western blots, and panel (**B**) shows results from four experiments assessed by digital densitometry; results (intensity) are expressed as the mean ± SEM. Panel (**C**) shows the effect of 50 pg/mL of Cdt containing wild-type CdtB (CdtB^WT^) or CdtB containing mutations (CdtB^R117A^, CdtB^R144A^, and CdtB^A163R^) on p21^CIP1/WAF1^ levels in MyLa cells. A representative Western blot is shown, as well as results from four experiments that were analyzed by digital densitometry; results are expressed as a percentage of p21^CIP1/WAF1^ levels observed in control (untreated) cells; * indicates statistical significance (*p* < 0.05) when compared to untreated cells.

To verify that the effects of Cdt on p21^CIP1/WAF1^ levels were not unique to the Jurkat cell line, the cutaneous T-cell lymphoma line, MyLa, was also assessed for altered p21^CIP1/WAF1^ levels when exposed to the same doses of Cdt. As shown in Figure 2A,B, MyLa cells treated with 0–400 pg/mL Cdt for 16 h exhibited significant increases in p21^CIP1/WAF1^levels: 4.5- (100 pg/mL Cdt) and 5.7-fold (400 pg/mL Cdt) over control levels. In addition to lymphoid cell lines, p21^CIP1/WAF1^ levels were assessed in primary human lymphocytes (HPBMCs). As shown in Figure 2A,B, exposure to 25 pg/mL Cdt resulted in a detectable but not statistically significant increase in p21^CIP1/WAF1^. Significant increases in p21^CIP1/WAF1^ levels were observed in the presence of 100 and 400 pg/mL Cdt leading to 361% ± 82% and 673% ± 185% over that observed in untreated control cells.

As noted earlier, we previously demonstrated that Cdt toxicity in lymphocytes (both primary and cell lines), macrophages, and mast cells is dependent upon PIP3 phosphatase activity exhibited by the active Cdt subunit, CdtB [24,25,26,27,28]. We generated and previously reported on the characterization of the enzymatic and toxic activities of Cdts containing targeted mutations within the CdtB subunit [24]. In each instance, we observed that the retention of lipid phosphatase activity, and not DNase activity, was a requisite for both Cdt-induced cell-cycle arrest and apoptosis in lymphocytes, as well as the induction of pro-inflammatory responses in macrophages. Therefore, we next determined the requirement for PIP3 phosphatase activity in Cdt-induced increases in the levels of p21^CIP1/WAF1^. Cdt containing CdtB mutant proteins that were previously described were employed [24]: CdtB^A163R^ retains lipid phosphatase activity, lacks DNase activity, and is toxic; CdtB^R144A^ exhibits low lipid phosphatase activity, exhibits increased DNase activity and is not toxic; CdtB^R117A^ exhibits low lipid phosphatase activity, retains DNase activity, and is not toxic. As shown in Figure 2C, elevations in p21^CIP1/WAF1^ levels were only observed when MyLa cells were treated with toxin containing the active wild-type subunit, CdtB^WT^ (2.5-fold increase), or the CdtB mutant, CdtB^A163R^ (3.2-fold increase) [24]. Cells exposed to CdtB mutant proteins that we previously demonstrated to be deficient in phosphatase activity and lack toxicity (CdtB^R144A^ and CdtB^R117A^) did not exhibit significant changes in levels of p21^CIP1/WAF1^: 1.2-fold and 1.7 fold, respectively. It is noteworthy that we previously reported that *A. actinomycetemcomitans* Cdt-induced cell-cycle arrest and apoptosis in human lymphocytes does not involve activation of the DNA damage response (DDR) [24,39]. These observations were confirmed as we now demonstrate that Cdt containing CdtB^WT^, as well as the other CdtB mutant proteins employed in this study, does not induce phosphorylation of the histone H2AX, a commonly employed indicator of DDR activation due to DNA damage (Figure S1, Supplementary Materials). 

The biologic activity of p21^CIP1/WAF1^ is governed by post-translational modifications such as phosphorylation; therefore, we also assessed Cdt-treated Jurkat cells for changes in phosphorylation status (T145; (pp21^CIP1/WAF1^)) (Figure 1C). Baseline levels of p21^CIP1/WAF1^ were very low, but detectable; as described above, these levels increased at 8–16 h in the presence of Cdt (see above). In contrast, the relative amount present as phosphorylated p21^CIP1/WAF1^ (pp21^CIP1/WAF1^) significantly declined in the presence of Cdt. Cells treated with 50 pg/mL Cdt for 16 h exhibited a reduction in the amount of pp21^CIP1/WAF1^ to 20.6% ± 3.4% of the amount observed in untreated control cells. Interestingly, the PI-3K signaling pathway exhibits cross-talk with other regulatory pathways; specifically, p21^CIP1/WAF1^ was shown to be a downstream target of activated Akt (pAkt) [40]. Furthermore, we showed that Cdt-treated cells exhibit reduced levels of pAkt (reduced kinase activity) [24]. These observations, along with our current findings that toxin-treated cells also exhibit reduced levels of pp21^CIP1/WAF1^, are consistent with the proposed molecular mode of action for CdtB which involves PIP3 phosphatase activity leading to PI-3K signaling blockade. To further support our findings and provide additional “proof-of-principle” evidence for a relationship between pAkt and p21^CIP1/WAF1^, we employed GSK690693, an Akt inhibitor [41]. Jurkat cells treated for 16 h with 0.5 μM GSK690693, exhibited a reduction in pp21^CIP1/WAF1^ to 0.7% ± 0.3% of that observed with untreated cells (Figure 1C).

### 2.2. Blockade of Cdt-Induced Increases in p21^CIP1/WAF1^ Results in Reduced Jurkat Cell Susceptibility to Cdt Toxicity

**Figure 3:**
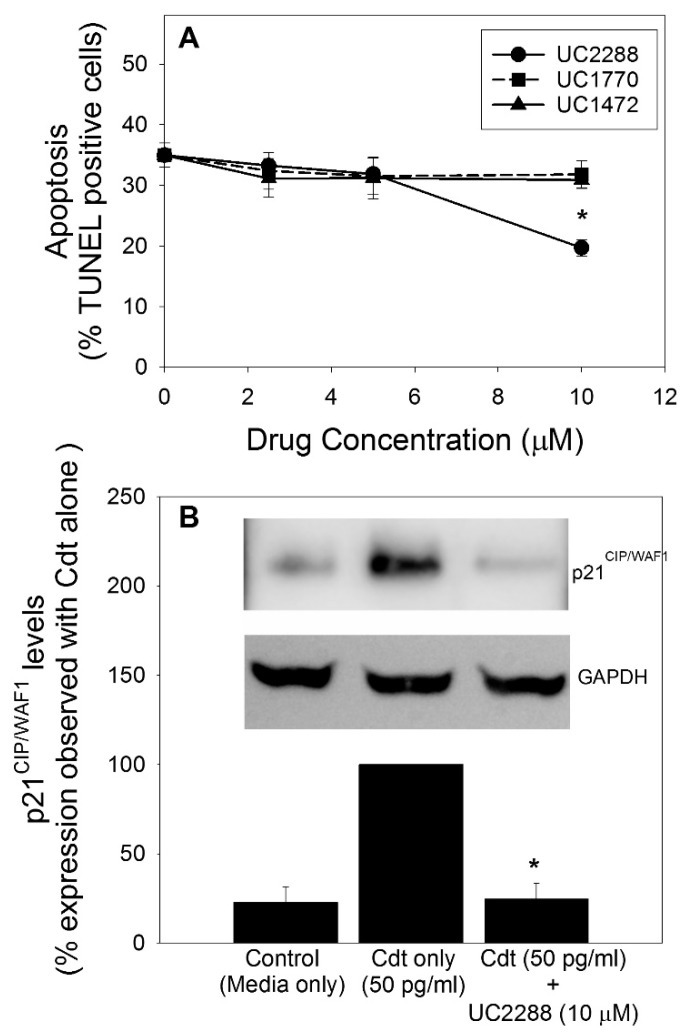
Effect of UC2288 on Cdt-induced apoptosis and on p21^CIP1/WAF1^ levels. Jurkat^WT^ cells were pre-incubated with 0–10 μM UC2288 or its inactive analogues UC1770 and UC1472 for 30 min. Cdt (50 pg/mL) was added to the cell cultures, and the cells were harvested 24 h later and analyzed for apoptosis using the TUNEL assay as described (panel (**A**)). Results are expressed as a percentage of apoptotic cells versus drug concentration; the mean ± SEM for four experiments is plotted. Panel (**B**) shows the effect of UC2288 on Cdt-induced p21^CIP1/WAF1^ levels at 16 h. A representative blot is shown along with results from four experiments. Western blots were analyzed by digital densitometry; the data are expressed as a percentage of the p21^CIP1/WAF1^ levels observed in cells treated with Cdt alone; * indicates statistical significance (*p* < 0.05) when compared to untreated cells.

We next extended our investigation to address the biological significance of Cdt-induced increases in p21^CIP1/WAF1^ by utilizing a two-pronged approach: pharmacologic modulation and altered expression using gene editing. The novel inhibitor of p21^CIP1/WAF1^, UC2288, was demonstrated to reduce p21^CIP1/WAF1^ levels [42]; therefore, we firstly employed UC2288 as a potential inhibitor of Cdt toxicity by virtue of its ability to block increases in p21^CIP1/WAF1^. As shown in Figure 3A, pre-treatment of Jurkat^WT^ cells with UC2288 (2.5–10 μM) resulted in reduced Cdt-induced apoptosis in the presence of the highest concentration of the inhibitor (10 μM); interestingly, this is the same concentration reported to be effective in other studies [42]. Untreated control cells exhibited 7.7% ± 1.1% terminal deoxynucleotidyl transferase (TdT)-mediated dUTP nick end labeling (TUNEL)-positive cells and, in the presence of 50 pg/mL Cdt, the percentage of TUNEL-positive cells increased to 35.0% ± 1.9%. Pre-treatment of cells with UC2288 reduced the percentage of TUNEL-positive cells to 19.7% ± 1.4%. Two inactive analogues of UC2288 were employed: UC1770 and UC1472; neither of these altered Cdt-induced apoptosis, as the percentage of TUNEL-positive cell was not reduced below 31% when cells were treated with equivalent concentrations of these analogues. The ability of UC2288 to block Cdt-induced increases in p21^CIP1/WAF1^ levels was also confirmed (Figure 3B). Results are expressed as a percentage of p21^CIP1/WAF1^ levels observed in cells exposed to toxin alone (100%); the latter represents almost a five-fold increase over control cells (medium only). Pre-treatment with 10 μM UC2288 resulted in a significant reduction of p21^CIP1/WAF1^ levels to 24.7% of that observed with cells treated with toxin alone.

**Figure 4:**
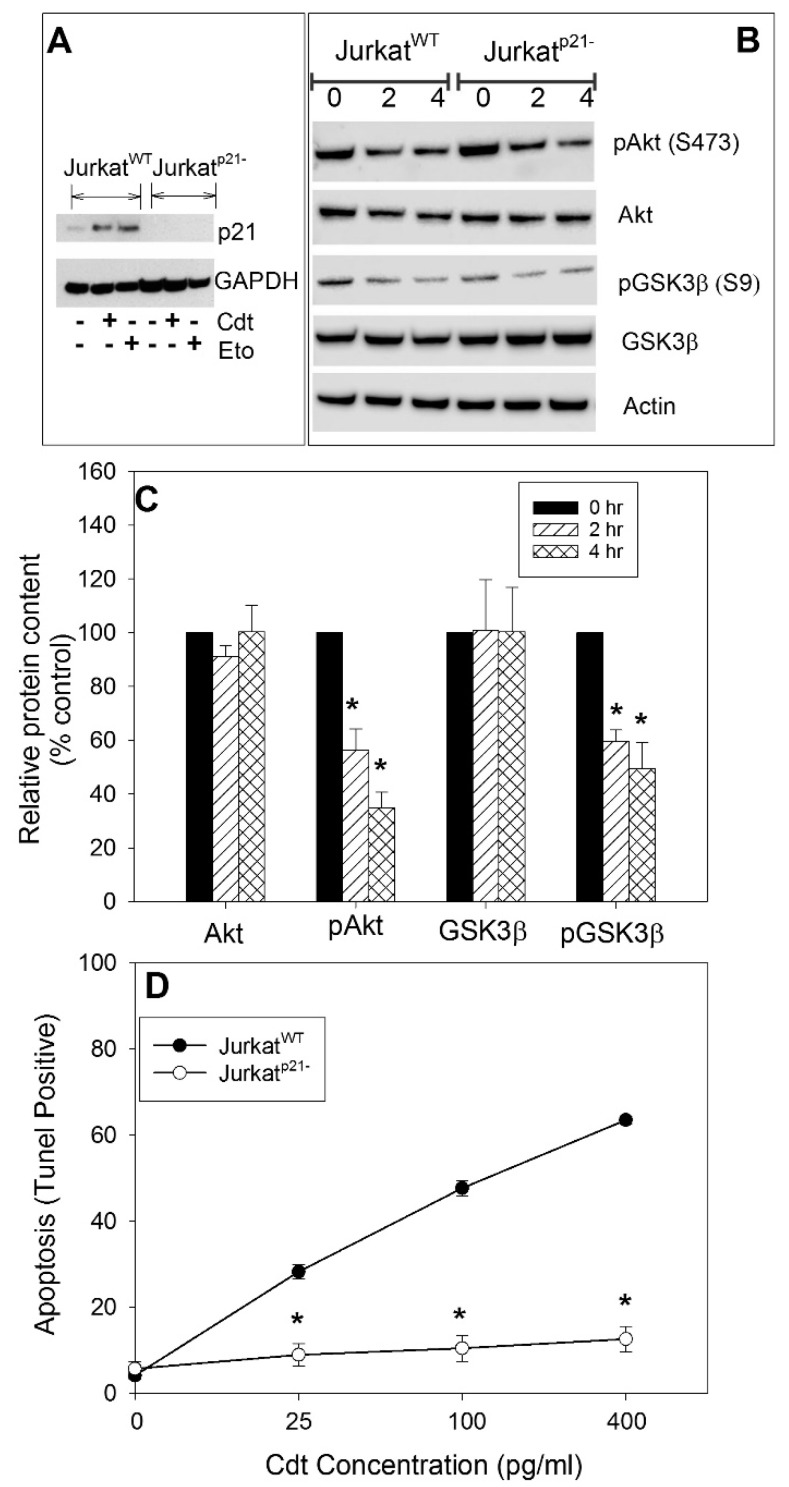
Analysis of Jurkat^p21−^ cells. CRISPR/Cas9 gene editing was employed to produce a Jurkat^p21−^ cell line. Panel (**A**) shows the results of p21^CIP1/WAF1^ analysis by Western blot of both Jurkat^WT^ and Jurkat^p21−^ cells at 16 h following exposure to either Cdt (50 pg/mL) or etoposide (Eto; 50 μM); GAPDH is shown as a gel loading control. Panels (**B**,**C**) show the effect of Cdt on PI-3K signaling targets. Jurkat^WT^ and Jurkat^p21−^ cells were treated with medium alone (0) or with Cdt (50 pg/mL) for 2 or 4 h and then analyzed by Western blot for the expression of Akt, pAkt (S473), GSK3β, and pGSK3β (S9), as well as actin which served as a loading control. Representative blots are shown in panel (**B**), and the results of three experiments for Jurkat^p21−^ cells are shown in panel (**C**). Data are plotted as the percentage of protein expressed in untreated control cells; the mean ± SEM is shown and * indicates statistical significance (*p* < 0.05) when compared to untreated cells. Panel (**D**) shows the effect of Cdt on apoptosis (TUNEL-positive) in Jurkat^WT^ and Jurkat^p21−^ cells. The percentage of apoptotic cells was determined at 24 h and is plotted versus Cdt concentration; the mean ± SEM is shown for three experiments; * indicates statistical significance (*p* < 0.01) when compared to untreated cells.

To confirm our observations with UC2288 and to better understand the biological significance of Cdt-induced elevations in p21^CIP1/WAF1^ levels, we utilized CRISPR/Cas9 gene editing to establish a stable Jurkat cell line deficient in p21^CIP1/WAF1^ expression (Jurkat^p21−^). As shown in Figure 4A, Jurkat^p21−^ cells were unable to express p21^CIP1/WAF1^ when challenged with either Cdt or etoposide for 16 h; in contrast, Jurkat^WT^ cells exhibited clear elevations in this protein when challenged with either agent under identical conditions. As noted above, we established that Cdt-induced toxicity is dependent on the CdtB subunit’s ability to function as a PIP3 phosphatase, thereby mediating blockade of the PI-3K signaling pathway [26,27]. Therefore, we next verified that Jurkat^p21−^ cells remained susceptible to toxin-induced signaling blockade. Specifically, cells were assessed after 2 and 4 h of exposure to Cdt for changes in the phosphorylation status of two downstream PI-3K signaling targets: Akt and GSK3β. Jurkat^WT^ and Jurkat^p21−^ cells were treated with 50 pg/mL Cdt and then analyzed by Western blot for the presence of Akt, pAkt, GSK3β, and pGSK3β at 0, 2, and 4 h. Figure 4B shows representative immunoblots indicating that the levels of both pAkt and pGSK3β were reduced at 2 and 4 h in both Jurkat^WT^ and Jurkat^p21−^; in contrast, the total amount of these proteins (Akt and GSK3β) remained unchanged. In previous studies, we reported that decreases in pAkt and pGSK3β within Jurkat^WT^ cells were statistically significant with levels of pAkt reduced to 48.7% ± 9.3% (2 h) and 45.5% ± 11.6% (4 h) relative to untreated control cells; likewise, pGSK3β levels were reduced to 55.7% ± 6.0% and 47.6% ± 6.1% of control values at 2 and 4 h, respectively [24]. Figure 4C shows similar compiled results from multiple experiments for Jurkat^p21−^ cells. Akt and GSK3β levels remained relatively constant for the 4-h period. In contrast, pAkt levels were reduced to 56.4% ± 19% (2 h) and to 34.8% ± 16.7% (4 h); pGSK3β levels were reduced to 59.7% ± 22.7% (2 h) and 49.5% ± 21.7% (4 h). 

Jurkat^WT^ and Jurkat^p21−^ cells were next assessed and compared for their susceptibility to Cdt-induced apoptosis using the TUNEL assay (Figure 4D) following a 24-h treatment with toxin. Consistent with our previous findings, Jurkat^WT^ cells exhibit dose-dependent apoptosis; the percentages of TUNEL-positive were 4.2% ± 0.9% (0 Cdt), 28.3% ± 1.6% (25 pg/mL Cdt), 47.7% ± 1.7% (100 pg/mL Cdt), and 63.5% ± 0.9% (400 pg/mL Cdt). In contrast, Jurkat^p21−^ cells were resistant to Cdt-induced apoptosis; cells incubated with 0–400 pg/mL toxin exhibited 5.7% ± 1.7%, 8.9% ± 2.6%, 10.5% ± 3.0%, and 12.6% ± 3.0% apoptotic cells. It is noteworthy that Jurkat^p21−^ cells retained the capacity to undergo apoptotic cell death as they remained sensitive to paclitaxel (Figure S2, Supplementary Materials). 

**Figure 5:**
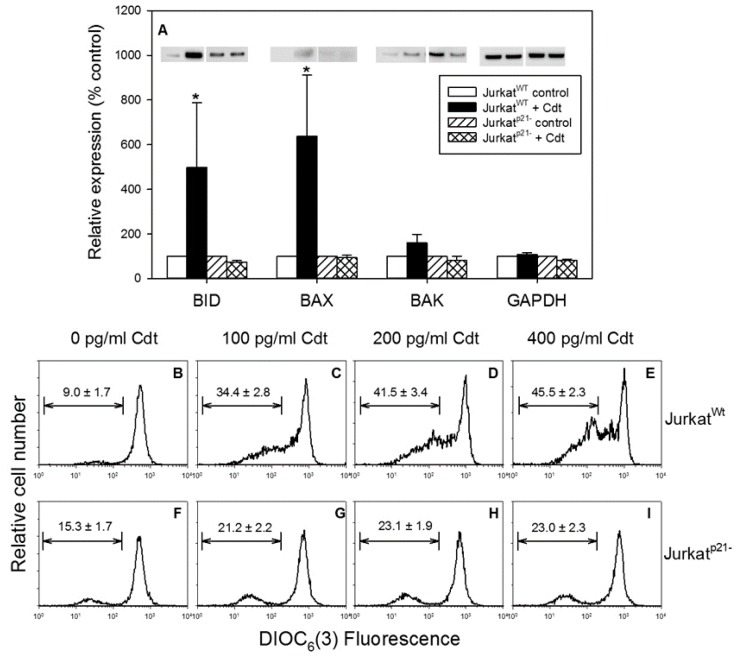
Effect of Cdt on the expression of pro-apoptotic Bcl-2 family members, as well as on the ΔΨm. Panel (**A**): Jurkat^WT^ and Jurkat^p21−^ cells were incubated with medium or 50 pg/mL Cdt for 8 h. Cells were then analyzed by Western blot for Bid, Bax, Bak, and GAPDH (loading control). Representative blots are shown on top for Bid, Bax, Bak, and GAPDH for Jurkat^WT^ cells exposed to medium and Cdt-treated, as well as for Jurkat^p21−^ cells exposed to medium and Cdt-treated. Western blots were analyzed by digital densitometry; results represent the levels of protein expressed as a percentage of that observed in respective control cells. The mean ± SEM for five experiments is shown; * indicates statistical significance (*p* < 0.05) when compared to untreated cells. Panels (**B**–**I**) show the effect of Cdt (0–400 pg/mL) on the ΔΨm in both Jurkat^WT^ (panels (**B**–**E**)) and Jurkat^p21−^ cells (panels (**F**–**I**)). ΔΨm was determined using DIOC_6_(3), and the percentage of cells exhibiting a reduction the membrane potential was determined using the analytical gate indicated; the numbers represent the mean ± SEM of three experiments, each performed in duplicate.

Previously, we demonstrated that Cdt-induced apoptosis involves the intrinsic apoptotic pathway and, in particular, development of the mitochondrial permeability transition (MPT) [39,43]. Therefore, we assessed and compared Jurkat^WT^ and Jurkat^p21−^ for expression of pro-apoptotic members of the Bcl-2 protein family in response to Cdt. Treatment of Jurkat^WT^ cells with 50 pg/mL Cdt resulted in significant increases in both Bid and Bax at 8 hrs (Figure 5A); Bid levels increased by 498.3% ± 290.5% relative to control cells and Bax levels increased by 637.0% ± 274.7%. A consistent, but not statistically significant, increase was observed for Bak to 160% ± 38.0%. In comparison, Cdt failed to induce increases in the levels of any of the three pro-apoptotic proteins in Jurkat^p21−^ cells; relative to untreated cells, Bid, Bax, and Bak expression levels were 73.2% ± 7.5%, 94.2% ± 10.5%, and 80.4% ± 20.4%, respectively.

Cdt was also assessed for its ability to induce the MPT in both Jurkat^WT^ and Jurkat^p21−^ cells by measuring a decline in the mitochondrial transmembrane potential (ΔΨm) using the fluorochrone DIOC_6_(3) [39,43]. As shown in Figure 5B–E, Jurkat^WT^ cells treated with 0–400 pg/mL Cdt exhibited a dose-dependent increase in the percentage of cells exhibiting a decline in the ΔΨm, with 9% in control cells versus 34.4%, 41.5%, and 45.5% in cells exposed to 100, 200, and 400 pg/mL Cdt. In contrast, Jurkat^p21−^ did not exhibit a significant change in the ΔΨm; 15.3% of control cells exhibited reduced ΔΨm, and Cdt treatment resulted in a slight, but not statistically significant increase to 21.2%, 23.1%, and 23% in the presence of 100, 200, and 400 pg/mL Cdt, respectively (Figure 5 F–I).

### 2.3. Cdt-Induced Increases in p21^CIP1/WAF1^ Levels and Induction of Apoptosis Are Blocked by the Chaperone HSP90

**Figure 6:**
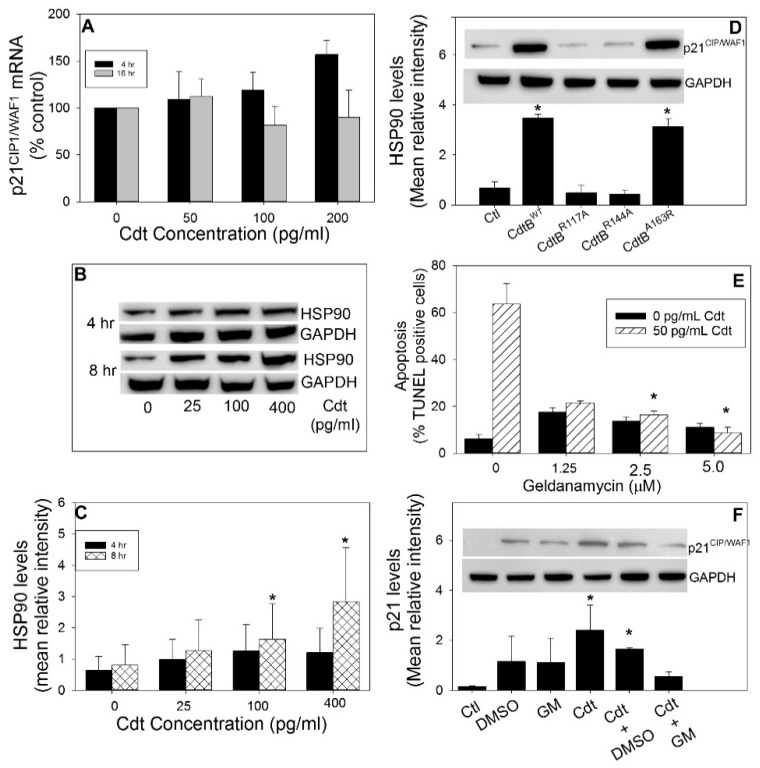
Cdt-induced increases in p21^CIP1/WAF1^ and induction of apoptosis are dependent upon HSP90. Panel (**A**) shows the effects of Cdt on p21^CIP1/WAF1^ messenger RNA (mRNA) by RT-PCR. Jurkat cells were incubated with 50 pg/mL Cdt for 4 and 16 h. RNA extraction, complementary DNA (cDNA) synthesis, RT-PCR, and changes in mRNA were calculated as previously described [28]. Results are the mean of three experiments plotted as a percentage of mRNA levels observed in control cells (time 0). Panels (**B**,**C**) show the results of experiments that assess the effect of Cdt on HSP90 levels. Jurkat cells were treated with Cdt (0–400 pg/mL) for 4 and 8 h, and the cell extracts were assessed by Western blot. Panel (**B**) shows a representative Western blot with GAPDH as a loading control. Panel (**C**) shows the results of four experiments; Western blots were analyzed by digital densitometry, and the results are plotted as the mean intensity ± SEM. Panel (**D**) shows Western blot analysis of the effect of 50 pg/mL Cdt containing CdtB^WT^ or CdtB containing mutations (CdtB^R117A^, CdtB^R144A^, and CdtB^A163R^) on HSP90 levels in Jurkat cells. A representative Western blot is shown, as well as results from three experiments that were analyzed by digital densitometry; results are expressed as the mean intensity ± SEM; * indicates statistical significance (*p* < 0.05) when compared to untreated cells. The effects of geldanamycin D (GM) on Cdt-induced apoptosis are shown in panel (**E**). Jurkat cells were incubated with 0–5 μM GM for 60 min, followed by the addition of medium and 50 pg/mL Cdt, before being analyzed for apoptosis (TUNEL assay) 24 h later. The percentage of TUNEL-positive cells is plotted as the mean ± SEM of four experiments. Panel (**F**) shows the effects of GM on Cdt-induced increases in Jurkat cell p21^CIP1/WAF1^ level. Jurkat cells were treated with medium or Cdt (50 pg/mL) in the presence of DMSO (vehicle) or 2.5 μM GM for 16 h. Cells were analyzed by Western blot and digital densitometry for the levels of p21^CIP1/WAF1^. A representative blot and the results from three experiments is shown; results are plotted as the mean ± SEM of three experiments; * indicates statistical significance (*p* < 0.05) when compared to untreated cells.

The intracellular levels of p21^CIP1/WAF1^ are controlled transcriptionally, as well as post-translationally, by the proteasome. Experiments were carried out to account for the mode of Cdt-induced increases in p21^CIP1/WAF1^. We firstly addressed the effect of Cdt on p21^CIP1/WAF1^ messenger RNA (mRNA) levels in Jurkat cells. As shown in Figure 6A, p21^CIP1/WAF1^ mRNA levels were marginally elevated at high toxin doses within 4 h and declined in the presence of the same doses of Cdt at 16 h; however, these changes were not statistically significant. It should also be noted that *A. actinomycetemcomitans* Cdt-induced increases in lymphocyte p21^CIP1/WAF1^ levels were previously shown to be p53-independent [35]. These findings were confirmed as we demonstrate that Cdt-induced p21^CIP1/WAF1^ increases were observed in the presence of the p53 inhibitor, pifithrin-α (Figure S3, Supplementary Materials); this inhibitor also failed to block Cdt-induced apoptosis. Similar results were observed with Molt-4 cells (data not shown). On the post-translational side, the chaperone HSP90 was shown to stabilize p21^CIP1/WAF1^ and prevent its degradation by the proteasome [44,45,46]. Jurkat cells were assessed for changes in the levels of HSP90 by Western blot following 4 and 8 h of exposure to Cdt (Figure 6B,C). Untreated cells exhibited detectable levels of HSP90 at both time points. Exposure of Jurkat cells to Cdt for 4 h resulted in 1.5- (25 pg/mL), 2.0- (100 pg/mL), and 1.9-fold (400 pg/mL) increases in HSP90 levels. Treatment with the same doses of Cdt for 8 h resulted in increases of 1.6-, 1.5-, and 3.6- fold. The dependence of Cdt-induced increases in HSP90 on CdtB-associated PIP3 phosphatase activity was also assessed (Figure 6D). Jurkat cells treated with Cdt containing the active CdtB subunits CdtB^WT^ and CdtB^A163R^ exhibited increased HSP90 levels of 5.1-fold and 4.6-fold over levels observed in control cells, respectively. The HSP90 levels in cells treated with the PIP3 phosphatase-inactive CdtB units, CdtB^R117A^ and CdtB^R144A^, did not change.

The relationship between HSP90 and Cdt-induced increases in p21^CIP1/WAF1^ levels, as well as the induction of apoptosis, was demonstrated by employing geldanamycin (GM), an inhibitor of HSP90 ATPase activity [47]. As shown in Figure 6E, GM was assessed for its ability to inhibit HSP90 and in turn Cdt-induced apoptosis. Jurkat cells were pre-treated with medium or GM (0–5 μM) for 1 h, followed by the addition of medium or Cdt (50 pg/mL). Cells were assessed for apoptosis (TUNEL-positive) 24 h later (Figure 6E). Cells treated with medium exhibited 6.2% ± 1.8% TUNEL-positive cells; in comparison, cells treated with 50 pg/mL Cdt exhibited 63.7% ± 8.8% TUNEL-positive cells. Pre-treatment of cells with GM followed by the addition of toxin resulted in a dose-dependent reduction in apoptosis. In the presence of 1.25, 2.5, and 5 μM GM, the percentage of TUNEL-positive cells was reduced to 21.6% ± 0.7%, 16.4% ± 1.7%, and 8.7% ± 2.3%. It should be noted that GM alone exhibited low levels of toxicity (net 5–10% over untreated cells).

In addition to evaluating the effects of GM on Cdt-induced apoptosis, Jurkat cells were also assessed for p21^CIP1/WAF1^ levels by Western blot (Figure 6F). Untreated Jurkat cells contained marginally detectable levels of p21^CIP1/WAF1^; cells treated with diluent (DMSO) or GM (2.5 μM) exhibited slight increases in p21^CIP1/WAF1^ to 1.2 ± 1.0 and 1.1 ± 0.98, respectively. Cells exposed to Cdt alone exhibited a significant increase in p21^CIP1/WAF1^ levels to 2.4 ± 1.0; pretreatment of cells with 2.5 μM GM reduced p21^CIP1/WAF1^ to near baseline levels (0.56 ± 0.19).

## 3. Discussion 

### 3.1. Functional Significance of Cdt-induced Increases in the Levels of p21^CIP1/WAF1^

We now report that *A. actinomycetemcomitans* Cdt induces intracellular increases of p21^CIP1/WAF1^ in human lymphocyte cell lines, as well as primary HPBMCs. These observations are consistent with those of other investigators who also demonstrated similar increases in response to Cdt derived from either *A. actinomycetemcomitans* or *Haemophilus ducreyi* [16,18,35,36]. P21^CIP1/WAF1^ increases were observed in a murine B-cell hybridoma cell line, fibroblasts, the Hep-2 carcinoma cell line, and a gingival squamous cell carcinoma cell line, Ca9-22. These investigators did not demonstrate the biological significance of the observed Cdt-dependent changes in cellular levels of p21^CIP1/WAF1^. As noted earlier, increases in p21^CIP1/WAF1^ levels are typically observed in response to cell stress or following DNA damage. The binding of p21^CIP1/WAF1^ to cyclins and their CDK binding partner (e.g., CDK1 or CDK2) results in inhibition of the kinase complex, resulting in cell-cycle arrest (reviewed in References [29,31,32,33]). It is generally accepted that p21^CIP1/WAF1^ regulates both cell-cycle arrest, i.e., checkpoint activation, and apoptosis. Collectively, the actions of p21^CIP1/WAF1^ promote cell survival and provide time for cells to undergo DNA repair before completing the cell cycle. However, it is becoming increasingly clear that the role of p21^CIP1/WAF1^ in regulating apoptosis is more complex than originally thought [30,48]. For example, several investigators demonstrated that pro-apoptotic agents induce increases in p21^CIP1/WAF1^ and, furthermore, over-expression of p21^CIP1/WAF1^ enhances apoptosis [49,50,51,52]. Likewise, cells and animals deficient in p21^CIP1/WAF1^ expression exhibit reduced susceptibility to apoptotic cell death [53,54]. 

In the current study, we employed a combination of gene editing (CRISPR/cas9) and pharmacologic intervention to assess the functional consequence of Cdt-induced increases in p21^CIP1/WAF1^. Collectively, our observations indicate that Cdt-induced apoptosis is dependent upon increased levels of p21^CIP1/WAF1^. Jurkat^p21−^ cells failed to exhibit apoptotic death following treatment with Cdt. Nonetheless, Jurkat^p21−^ cells retained their susceptibility to other pro-apoptotic agents such as paclitaxel. Additionally, we employed the novel p21^CIP1/WAF1^ inhibitor UC2288 whose structure is based upon sorafenib, another inhibitor of p21^CIP1/WAF1^. UC2288 is a new-generation inhibitor that exhibits greater selectivity with higher potency to attenuate p21^CIP1/WAF1^ levels [41,55]. Similar to our findings with Jurkat^p21−^ cells, Jurkat^WT^ cells treated with Cdt in the presence of 10 μM UC2288 also failed to exhibit increased levels of p21^CIP1/WAF1^; moreover, the drug suppressed Cdt-induced apoptosis. It should also be noted that the effective dose of UC2288 used in this study was identical to that employed in other studies [41,55].

**Figure 7:**
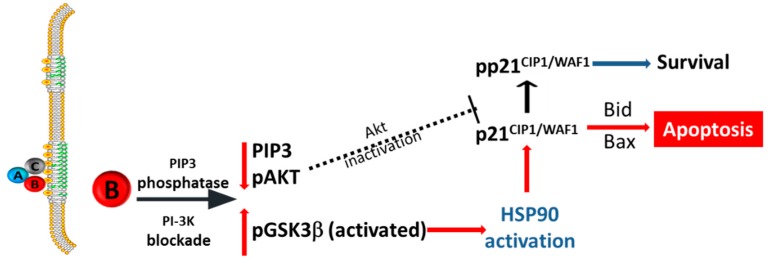
Overview of the proposed mechanism via which Cdt induces p21^CIP1/WAF1^-dependent apoptosis. Based upon the data presented in this paper, the proposed pathway of Cdt-induced p21^CIP1/WAF1^-dependent apoptosis is shown with red arrows. The dotted line shows the proposed relationship between Akt and the putative downstream target, p21^CIP1/WAF1^.

Our results demonstrate a pivotal role for p21^CIP1/WAF1^ in Cdt-induced apoptosis (Figure 7); these observations encompass a non-conventional role for p21^CIP1/WAF1^ as it proposes a pro-apoptotic function when increases in this regulatory protein are commonly thought to, at least initially, serve a pro-survival role. It is well established that the pleiotropic effects of p21^CIP1/WAF1^ are regulated by post-transcriptional modification. For example, phosphorylation of p21^CIP1/WAF1^ is known to alter its function which includes a shift in the balance between its pro- and anti-apoptotic effects [29,31,56,57]. In this regard, p21^CIP1/WAF1^ was shown to serve as a downstream target for phosphorylation (pp21^CIP1/WAF1^ (T-145)) by pAkt, the active form of Akt [58,59]. Consistent with these findings is our observation that Cdt-induced increases in p21^CIP1/WAF1^ were not only associated with the induction of apoptosis but also accompanied by a substantial shift in the distribution of pp21^CIP1/WAF1^ levels relative to untreated cells. It is noteworthy that, in addition to inducing p21^CIP1/WAF1^ increases, we previously established that treatment of lymphocytes with *A. actinomycetemcomitans* Cdt results in blockade of the PI-3K signaling cascade and, furthermore, Cdt toxicity is dependent upon perturbation of these early signaling events. Cdt-mediated PI-3K blockade is the result of potent lipid phosphatase activity, specifically PIP3 phosphatase activity, associated with the active Cdt subunit, CdtB [26]. Cells exposed to Cdt exhibit PI-3K signaling blockade within 2 h; this is characterized by PIP3 depletion and reduced Akt activity [24,27,28]. It is important to note that Jurkat^p21−^ cells retained their susceptibility to Cdt-induced PI-3K signaling blockade but were unable to undergo apoptosis due to their inability to produce p21^CIP1/WAF1^. We propose that depletion of pAkt in Cdt-treated cells limits phosphorylation of p21^CIP1/WAF1^ (Figure 7); this relationship is further supported by our finding that the Akt inhibitor, GSK690693, also depletes Jurkat^WT^ cells of pp21^CIP1/WAF1^. Collectively, these findings support a critical pro-apoptotic role for non-phosphorylated p21^CIP1/WAF1^ in mediating Cdt-induced cell death.

Finally, the link between increased p21^CIP1/WAF1^ levels and the onset of apoptosis was established in experiments in which we assessed the ability of Cdt to alter the expression of pro-apoptotic members of the Bcl-2 family. In previous studies, we, and others, established that Cdt-induced apoptosis involves development of the mitochondrial permeability transition state characterized by a decrease in the transmembrane potential and an increase in production of reactive oxygen species [43,60,61]; these events were followed by activation of the caspase cascade. Of particular relevance to our current study were our previous findings that over-expression of Bcl-2 blocked Cdt-induced apoptosis [39]. In this context, the pro-apoptotic requirement for p21^CIP1/WAF1^ in Cdt-induced apoptosis is further supported by our current observation that the toxin induces p21^CIP1/WAF1^-dependent upregulation of the pro-apoptotic proteins Bid, Bax, and Bak. P21^CIP1/WAF1^-dependency was demonstrated by the observation that the levels of these proteins were not altered in Cdt-treated Jurkat^p21−^ cells. Moreover, Jurkat^p21−^ cells also failed to exhibit changes in the ΔΨm as we observed with Cdt-susceptible cells. It is noteworthy that Gogada et al. [62] demonstrated that p21^CIP1/WAF1^ played a critical role in circumin-induced apoptosis by altering mitochondrial permeability, thereby facilitating the release of cytochrome c.

### 3.2. Cdt Toxicity Requires HSP90-Dependent Increases in the Levels of p21^CIP1/WAF1^

Eukaryotic cell-cycle progression and cell survival are regulated at multiple checkpoints involving a number of critical regulatory proteins. One such regulatory protein is p21^CIP1/WAF1^ which functions as a cyclin-dependent kinase inhibitor [29]. Elevated levels of p21^CIP1/WAF1^ are critical to its function; typically, p21^CIP1/WAF1^ is expressed at low levels under normal growth conditions as an unstable protein with a short half-life. It is well established that p21^CIP1/WAF1^ can be upregulated via a number of p53-dependent and -independent mechanisms (reviewed in Reference [31]). Likewise, Cdt-induced p21^CIP1/WAF1^ increases were reported to be both p53-dependent and -independent (reviewed in Reference [1]); these findings led others to propose that the role of p53 with respect to Cdt is dependent upon both the specific target cell and the source of toxin. Of particular relevance to this study, Sato et al. [35] demonstrated that *A. actinomycetemcomitans* Cdt-treated lymphocytes exhibit p53-independent increases in p21^CIP1/WAF1^. We confirmed these observations by demonstrating that *A. actinomycetemcomitans* Cdt-treated lymphocytes exhibited increases in p21^CIP1/WAF1^ and apoptosis when pre-exposed to the p53 inhibitor pifithrin-α.

Stress and/or DNA damage are known to induce increases in p21^CIP1/WAF1^ levels as a result of elevated transcription, RNA stability and/or decreased proteasomal degradation (reviewed in References [29,33]). Cdt-induced increases in p21^CIP1/WAF1^ were not accompanied by significant changes in mRNA levels. These findings led us to explore the possibility that the observed increases were instead due to protein stabilization and reduced proteasomal degradation. It is in this context that we considered the role of the heat shock protein HSP90 in both Cdt-induced increases in p21^CIP1/WAF1^ and toxicity. As noted above, p21^CIP1/WAF1^ is typically expressed at low levels as an unstable protein with a short half-life in normally growing cells. HSP90 is known to function as a chaperone that is critical to stabilizing proteins involved in several cellular processes [46]. In particular, HSP90 was shown to be recruited to p21^CIP1/WAF1^ where it binds via the WAF1/CIP1 stabilizing protein 39 (WISp39). The complex of HSP90, WISp39, and p21^CIP1/WAF1^ was reported to stabilize and protect the cell-cycle inhibitor from proteasomal degradation [44,45]. 

We extended these observations to Cdt-induced stress in lymphocytes by firstly assessing toxin-treated Jurkat cells for changes in both WISp39 and HSP90. Jurkat cells constitutively express WISp39, and we observed that exposure to Cdt did not result in further increases (data not shown). In contrast, Cdt-treated cells exhibited significant increases in HSP90 levels within 4 h, prior to the peak elevation in p21^CIP1/WAF1^. It should also be noted that the ability of CdtB to alter HSP90 levels, as demonstrated for p21^CIP1/WAF1^, was found to be dependent upon PIP3 phosphatase activity, since toxin-containing CdtB subunit mutants that lacked phosphatase activity were unable to induce increases in HSP90 levels. To further investigate the relationship between HSP90, p21^CIP1/WAF1^, and apoptosis, we employed the HSP90 inhibitor, GM. Specifically, our observations demonstrate that cells pre-treated with GM prior to exposure to Cdt prevented toxin induces increases in p21^CIP1/WAF1^; moreover, these cells exhibited decreased susceptibility to toxin-induced apoptosis, demonstrating a linkage between HSP90, changes in p21^CIP1/WAF1^ levels, and toxicity.

## 4. Conclusions

In summary, this study advances our understanding of the molecular events leading to *A. actinomycetemcomitans* Cdt-mediated toxicity in lymphocytes. We previously demonstrated that Cdt toxicity is dependent upon PI-3K signaling blockade in lymphocytes, mast cells, and macrophages [2,24,25,26,28,63,64]. A key to impairment of this signaling pathway is the ability of the active Cdt subunit, CdtB, to function as a PIP3 phosphatase. Our current observations utilized both pharmacologic and gene editing approaches to demonstrate that toxin-induced apoptosis is also dependent upon increased levels of p21^CIP1/WAF1^. Furthermore, toxin-induced increases in this critical regulatory protein are dependent upon HSP90. Moreover, the timeline for these and previous observations suggest a sequence in which the earliest events involve: Cdt binding to membrane cholesterol via CdtC and CdtB, internalization of CdtB, and depletion of PIP3, leading to a concomitant decrease in pAkt (loss of activity), increased expression/activation of HSP90, increased levels of p21^CIP1/WAF1^, and increased levels of pro-apoptotic Bcl-2 family proteins [28,65]. We propose that the ability of Cdt to impair lymphocyte proliferation and promote cell death therefore compromises the host response to Cdt-producing organisms. Our observations are of particular significance, as Cdts are produced by not only *A. actinomycetemcomtans*, but also by over 30 γ- and ε- Proteobacteria [1,2]. Therefore, we further propose that the action of this putative virulence factor contributes to the pathogenesis of a range of diseases leading to persistent infection by Cdt-producing pathogens. Our current findings not only contribute to a greater understanding of the molecular events critical to Cdt toxicity, but also provide avenues for developing novel approaches to attenuating the immunoinhibitory effects of the toxin.

## 5. Materials and Methods

### 5.1. Reagents and Antibodies

We previously reported on the construction and expression of the plasmids which contain the *cdt* genes for the holotoxin (pUCAacdtABC^his^), as well as those constructs containing CdtB mutations [10]. The histidine-tagged holotoxin was isolated by nickel affinity chromatography as previously described [66]. All antibodies were obtained from commercial sources as indicated. UC2288 was provided by RH Weiss), GM was purchased from Thermofisher Scientific (Waltham, MA, USA), and GSK690962 was purchased from Cayman Chemical (Ann Arbor, MI, USA). 

### 5.2. Culture Conditions and CRISPR/cas9-Mediated Genome Editing

Two human lymphoid cell lines were employed in these studies: the T-cell leukemia cell line Jurkat (E6-1) and the cutaneous T-cell lymphoma cell line, MyLa2059. Cells were maintained as previously described [10]. Jurkat^WT^ cells were cultured in Roswell Park Memorial Institute (RPMI) 1640 supplemented with fetal bovine serum (FBS) (10%), glutamine (2 mM), 4-(2-hydroxyethyl)-1-piperazineethanesulfonic acid (HEPES) (10 mM), penicillin (100 U/mL), and streptomycin (100 μg/mL). MyLa2059 cells were maintained in the same medium containing 20% FBS. Human peripheral blood mononuclear cells (HPBMCs) were prepared and incubated as described previously [67]; blood was obtained using an Institutional Review Board-approved protocol, and all donors provided written consent. 

To generate p21^CIP1/WAF1^-deficient Jurkat cells (Jurkat^p21−^), we utilized CRISPR/cas9 technology (Santa Cruz Biotechnology; Santa Cruz, CA, USA) as previously described [68]. Cells were transfected (Amaxa Nucleofector system; Lonza, Basel) with a pool of three plasmids, each encoding the Cas9 nuclease and a p21^CIP1/WAF1^-specific 20-nt guide RNA (gRNA). Cells were co-transfected with a pool of three plasmids each containing a homology-directed DNA repair (HDR) template; this corresponded to sites generated by the p21^CIP1/WAF1^ CRISPR/cas9 knockout plasmids. HDR plasmids insert the puromycin resistance gene that facilitates selection of stable knockout cells. Cells were firstly incubated for a five-day period following transfection and then for an additional seven days in incubation in puromycin (5 μg/mL). Limiting dilution of surviving cells was utilized to clone cells; clones were expanded and assessed by Western blot analysis for both the presence of p21^CIP1/WAF1^ their ability to increase p21^CIP1/WAF1^ levels in response to Cdt or etoposide. Clones determined to be deficient in p21^CIP1/WAF1^ were then cloned a second time using limiting dilution (Figure 4A). Jurkat^p21−^ cell lines were maintained in medium containing puromycin (1 μg/mL); experiments were conducted in medium without puromycin. 

### 5.3. Assessment of Apoptosis

Jurkat^WT^ and Jurkat^p21−^ cells were challenged with Cdt or medium (control) for 24 h, and apoptosis was assessed by measuring DNA fragmentation (In Situ Cell Death Detection Kit; Sigma Aldrich, St. Louis, MI, USA) [43]. Briefly, after 24 h of incubation, the cells were re-suspended in freshly prepared 4% formaldehyde and permeabilized with 0.1% Triton X-100 for 2 min at 4 °C; then, they were washed and stained with a solution containing FITC-labeled nucleotide and terminal deoxynucleotidyl transferase (TdT). Flow cytometry was employed to measure FITC fluorescence with a laser at 488 nm to excite the fluorochrome; fluorescence emission was measured through a 530/30-nm bandpass filter.

To measure development of the permeability phase transition, Jurkat cells were incubated for 18 h in medium alone or containing Cdt under conditions described above. Changes in the transmembrane potential (ΔΨ_m_) were determined using 4 nM 3,3′-dihexyloxacarbocyanine (DIOC_6_(3); Thermofisher) [39,43]. Cells were stained for 15 min (37 °C) with the fluorochrome and fluorescence measured following excitation with a laser at 488 nm (250 mW), and emission was monitored through a 530/30-nm bandpass filter; at least 10,000 cells were analyzed per sample.

### 5.4. Western Blot Analysis

Cells were incubated with and without Cdt as described above; following the indicated incubation period, cells were solubilized in 20 mM Tris-HCl buffer (pH7.5) containing 150 mM NaCl, 1 mM EDTA, 1% NP-40, 1% sodium deoxycholate, and a protease inhibitor cocktail (ThermoFisher Scientific; Waltham, MA, USA). Samples (30 μg) were fractionated on 12% SDS-PAGE and then blotted onto PVDF membranes; the membranes were blocked with BLOTTO and then incubated with one of the following primary antibodies (Cell Signaling Technology; Danvers, MA, USA) for 18 h at 4 °C [12]: anti-Akt, anti-pAkt (S473), anti-GSK3β, anti-pGSK3β (S9), or anti-GAPDH; anti-p21^CIP1/WAF1^, anti-pp21^CIP1/WAF1^, and anti-HSP90 antibodies were also employed (Abcam; Cambridge, MA, USA). The membranes were incubated with goat anti-rabbit immunoglobulin conjugated to horseradish peroxidase (Southern Biotech Technology; Birmingham, AL, USA) after they were blocked and washed. The Western blots were developed using chemiluminescence and analyzed by digital densitometry (Li-Cor Biosciences; Lincoln, NE, USA) as previously described [25].

### 5.5. Statistical Analysis

The mean ± standard error of the mean was calculated for replicate experiments. Significance was determined using a Student’s *t*-test using SigmaPlot Software (Systat; San Jose, CA); a *p*-value of less than 0.05 was considered to be statistically significant.

## Supplementary Materials

The following are available online at https://www.mdpi.com/2076-0817/9/1/38/s1: Figure S1: Effect of Cdt containing CdtB^WT^ or one of the CdtB mutants on phosphorylation of H2AX; Figure S2: Susceptibility of Jurkat^p21−^ cells paclitaxel; Figure S3: Effect of pifithrin-α (PFT) on Cdt-induced increases in p21^CIP1/WAF1^ and apoptosis.
