# Supplementary material for: The Cell-Cycle Regulatory Protein p21CIP1/WAF1 Is Required for Cytolethal Distending Toxin (Cdt)-Induced Apoptosis
Source: Pathogens. 2020 Jan 2;9(1):38. doi: 10.3390/pathogens9010038 (PMC7168616; doi:10.3390/pathogens9010038)
Supplement: Supplementary file 1 [file pathogens-09-00038-s001.pdf]

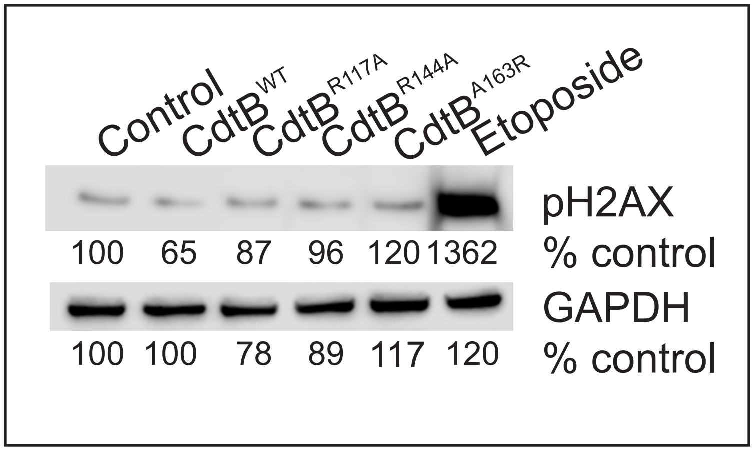

Figure S1: Effect of Cdt containing CdtB^{WT} or one of the CdtB mutant proteins on phosphorylation of H2AX. Jurkat^{WT} cells were incubated in the presence of 100 pg/ml Cdt containing either CdtB^{WT} or a mutant CdtB protein for 4 hr. Cells were then fractionated and analyzed by Western blot for the presence of pH2AX. A representative blot of three experiments is shown. Numbers represent the amount of pH2AX expressed as a percentage observed in control cells incubated in medium alone. Cells treated with etoposide (25 uM) were included as a positive control for H2AX phosphorylation.

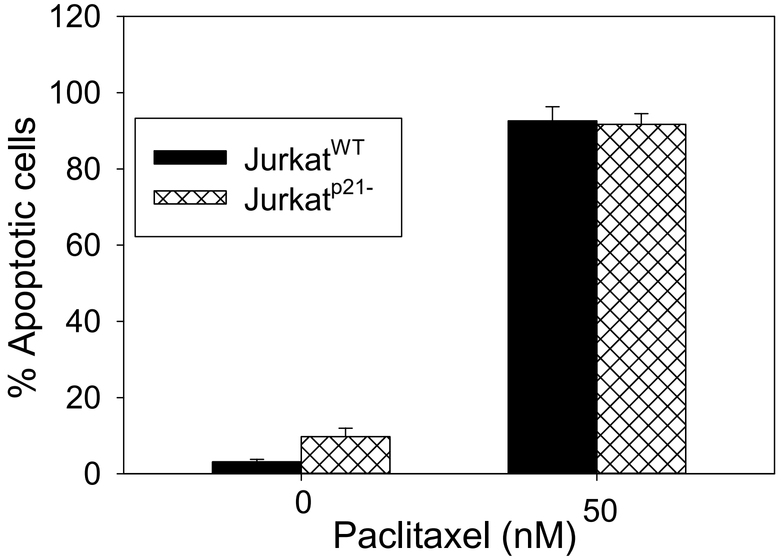

Figure S2: Susceptibility of Jurkat^{p21-} cells to paclitaxel. Jurkat^{WT} and Jurkat^{p21-} were incubated with 50 nM paclitaxel for 48 hr. The cells were then analyzed for apoptosis using the TUNEL assay and flow cytometric analysis. Data from three experiments are plotted as the percent of apoptotic cells (mean±SE).

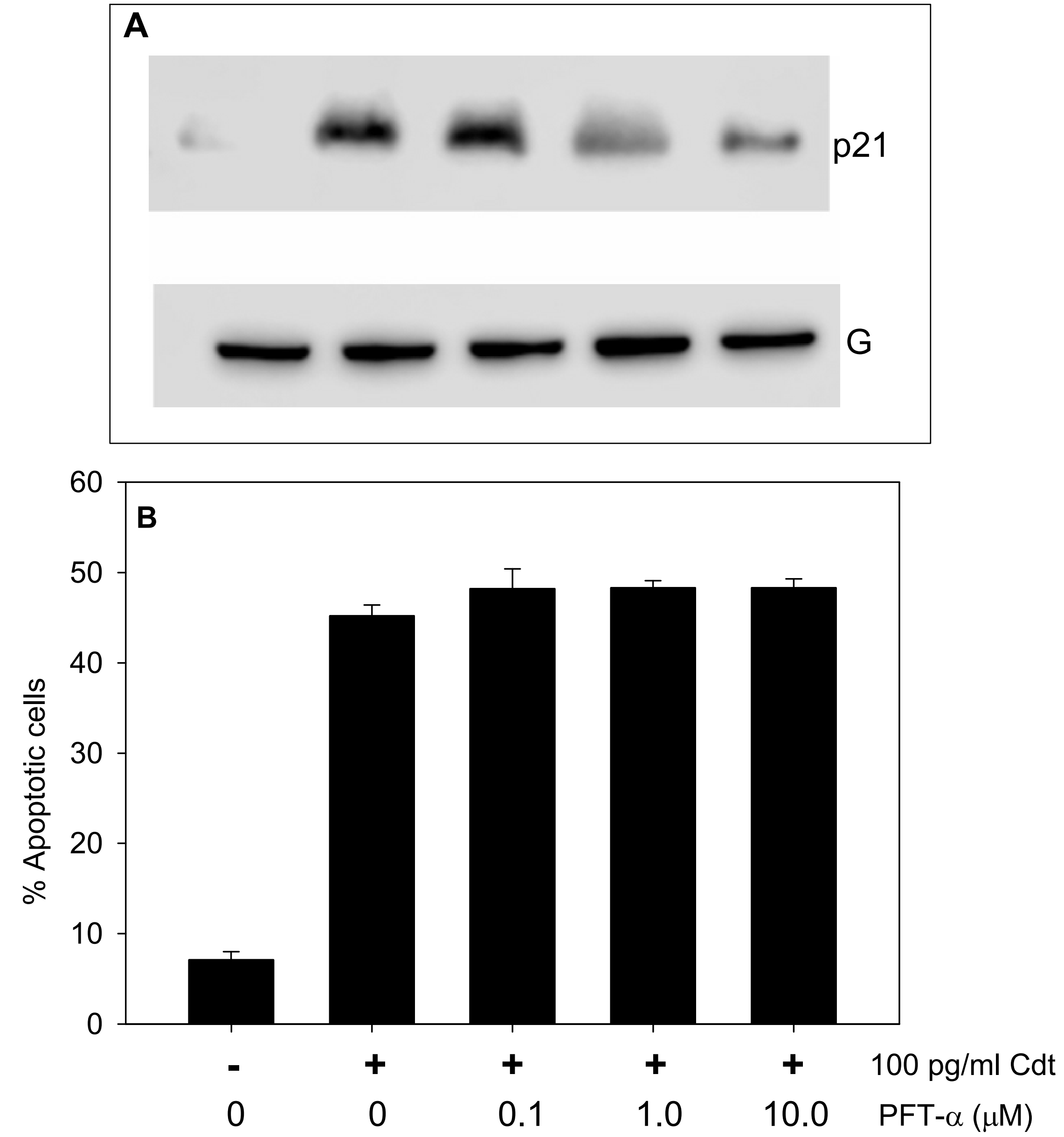

Figure S3: Effect of pifithrin-α (PFT) on Cdt-induced increases in $p21^{CIP1/WAF1}$ and apoptosis. $Jurkat^{WT}$ cells were pretreated with 0-10 μM PFT for 60 min followed by the addition of 100 pg/ml Cdt. Cells were analyzed for $p21^{CIP1/WAF1}$ levels by Western blot at 16 hr (panel A) and for apoptosis at 24 hr (panel B). The Western blot is representative of three experiments; bands showing $p21^{CIP1/WAF1}$ and GAPDH (G) are of (left to right) cells treated with medium alone, Cdt alone and Cdt in the presence of 0.1, 1.0 and 10.0 μM PFT-α. Apoptosis (TUNEL assay) results represent the mean±SD of three experiments.
